# Comparative Transcriptomic Analysis of Gene Expression Inheritance Patterns Associated with Cabbage Head Heterosis

**DOI:** 10.3390/plants10020275

**Published:** 2021-01-31

**Authors:** Shengjuan Li, Charitha P. A. Jayasinghege, Jia Guo, Enhui Zhang, Xingli Wang, Zhongmin Xu

**Affiliations:** 1 College of Horticulture, Northwest A&F University, Yangling 712100, China; li2016050305@nwafu.edu.cn (S.L.); berylgj8353@nwsuaf.edu.cn (J.G.); 2008117366@nwafu.edu.cn (E.Z.); wangxingli@nwafu.edu.cn (X.W.); 2 Department of Agricultural, Food and Nutritional Science, University of Alberta, Edmonton, AB T6G 2P5, Canada; charitha.jayasinghege@canada.ca

**Keywords:** *Brassica oleracea* L. var. capitata, heterosis, transcriptomics, allele-specific expression, cis-and trans-regulation

## Abstract

The molecular mechanism of heterosis or hybrid vigor, where F1 hybrids of genetically diverse parents show superior traits compared to their parents, is not well understood. Here, we studied the molecular regulation of heterosis in four F1 cabbage hybrids that showed heterosis for several horticultural traits, including head size and weight. To examine the molecular mechanisms, we performed a global transcriptome profiling in the hybrids and their parents by RNA sequencing. The proportion of genetic variations detected as single nucleotide polymorphisms and small insertion–deletions as well as the numbers of differentially expressed genes indicated a larger role of the female parent than the male parent in the genetic divergence of the hybrids. More than 86% of hybrid gene expressions were non-additive. More than 81% of the genes showing divergent expressions showed dominant inheritance, and more than 56% of these exhibited maternal expression dominance. Gene expression regulation by cis-regulatory mechanisms appears to mediate most of the gene expression divergence in the hybrids; however, trans-regulatory factors appear to have a higher effect compared to cis-regulatory factors on parental expression divergence. These observations bring new insights into the molecular mechanisms of heterosis during the cabbage head development.

## 1. Introduction

The phenomenon where offspring of genetically diverse parents show superior or beneficial alterations of agronomic traits, such as growth potentials, yield, fertility, or stress tolerance, compared to their parents, is known as heterosis or hybrid vigor [1]. Plant breeding programs widely benefit from this phenomenon in obtaining desired or improved crop qualities [2]. Cabbage (*Brassica oleracea* L. var. *capitata*) is one of the most consumed leafy vegetables in the world [3]. Since most of the commercial cultivars of cabbage are F1 hybrids obtained by outcrossing, understanding the underlying mechanisms of heterosis can help increase cabbage breeding efficiencies and improve the market qualities of cabbage, such as head size and appearance. 

Two classical genetic models, “dominance” and “overdominance”, have been widely discussed as potential genetic effects of heterosis [1,4]. According to the dominance model, complementation of multiple deleterious recessive alleles of each parent by superior, dominant alleles of the other parent results in heterosis. The overdominance model describes heterosis as a result of synergistic interactions between alleles at heterozygous loci of a hybrid. Nevertheless, these models can only explain single-locus heterosis [5]. Since most of the traits correlated to heterosis are controlled by multigenic effects, perspectives of energy-use efficiency and protein metabolism are commonly used to explain general multigenic heterosis [6,7,8]. Also, transcriptomic analysis has become a valuable tool that can be used to broaden our understanding of heterosis. For example, differentially expressed genes (DEGs) identified by transcriptomics can be used to predict crucial physiological pathways [9]. The data can also be used to identify genetic variations, including single nucleotide polymorphisms (SNPs) and small insertions/deletions (InDels) as well as imbalanced contributions of the parental genomes in the hybrid gene expression [10,11]. 

Gene expression divergence between hybrid and parents can be classified into multiple gene expression inheritance patterns. In additive expression inheritance, the transcript level of a given gene in a hybrid is similar to mid-parent. In non-additive inheritance, the hybrid transcript level can be similar to the parent showing the highest or lowest expression (high or low parent dominance; also referred to as positive or negative non-additivity), or it can be higher or lower than both parents (transgressive-up and transgressive-down; also referred to as overdominant and underdominant expression levels) [12,13,14]. All these expression inheritance patterns may be found in a single hybrid and can contribute to heterosis in different proportions [9]. For example, an expressed sequence tag (EST) microarray analysis in the F1 hybrids of maize showed the presence of all possible expression patterns; however, 78% of the differentially expressed ESTs exhibited additive expressions [12]. In *Nicotiana tabacum* L. hybrids, paternal expression level dominance was the most prominent, but 13 key genes regulating nicotine anabolism and transport showed transgressive-up or -down regulations [15]. 

Allele-specific regulation of gene expressions during environmental and stress responses indicates that allelic variations may play a fundamental role in the heterosis [16]. Allelic differences are widespread in hybrids and can have significant effects on gene expressions [17]. Parental alleles interacting with each other can result in cis- and trans-regulations. The cis-effects, such as the changes in promoters and enhancers, affect gene expression in a single chromosome and therefore are restricted to a chromosome from one parent. In contrast, the effects of trans-regulations, such as the changes in transcription factors, can affect chromosomes from both parents [18]. Even though both cis- and trans-regulations contribute to divergent gene expressions, their respective contributions are not clear. For example, in maize, allelic cis-regulatory variation between some inbred lines largely contributes to the gene expression divergence in the F1 hybrids [19]. In *Cirsium arvense*, the trans-effect shows a greater correlation with the parental expression divergence and tends to drive the higher expressions of paternal alleles [20]. In the F1 hybrids between *Arabidopsis thaliana* and *Arabidopsis arenosa*, both cis- and trans-regulations appear to mediate gene-expression divergence and chromatin modifications [21]. 

While heterosis is essential in cabbage breeding, previous studies of cabbage have mostly focused on phenotypes over underlying mechanisms [22,23]. Among the few studies evaluating genetic mechanisms, several have examined quantitative trait loci associated with the head shape or other quality-related traits [24,25]. Microarray-based analysis has also been used to search for potential regulatory genes related to heterosis [26]. However, none of these studies reflect the comprehensive gene expression changes between cabbage hybrids and their parents. In this study, we examined horticultural traits and transcriptome profiles of four F1 cabbage hybrids and their parents. We evaluated the genetic and gene expression divergences between hybrids and parents to understand the genetic regulatory mechanisms and their contribution to heterosis. Cellular processes represented by DEGs, transcription factors associated with cabbage head growth and development, and the cis- and trans-effects were investigated. Our research provides a comparative perspective on the gene expression inheritance patterns between cabbage hybrids and their parents. 

## 2. Results

### 2.1. Cabbage Hybrids Show Heterosis in Several Horticultural Traits

**Figure 1:**
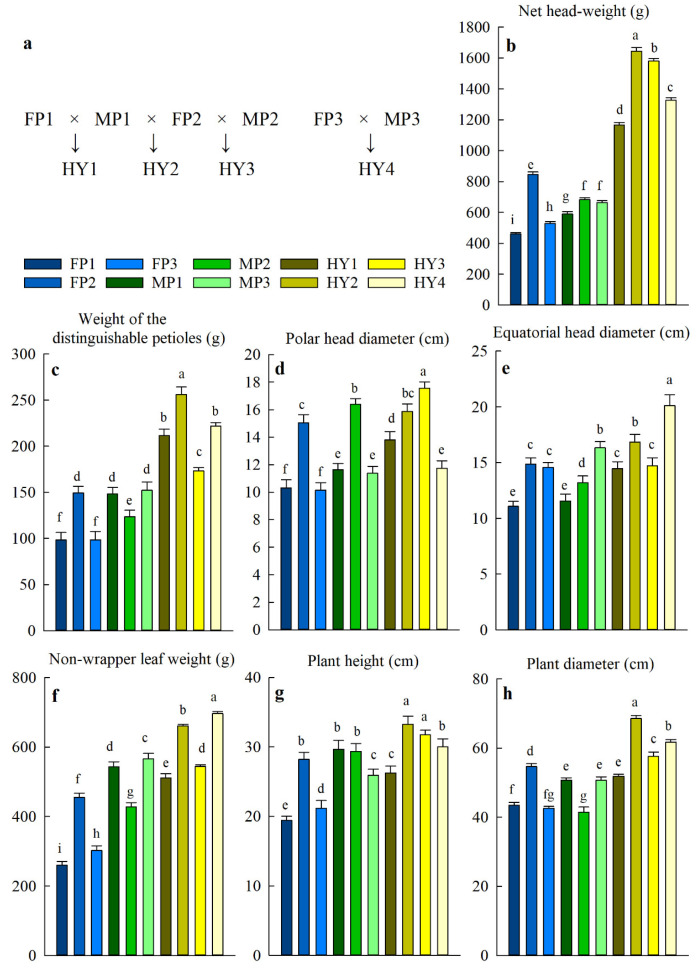
The crosses used for the generation of hybrids and the differences in the cabbage head and plant size traits. (**a**) The female parent (FP) and the male parent (MP) crosses used to obtain different cabbage hybrids (HY). HY1 and HY2 share the MP; HY2 and HY3 share the FP; HY4 does not share parents with any other hybrid. (**b**–**h**) Variation of cabbage head size and plant size among cabbage hybrids and parents. Data are means ± SD, n = 3 blocks, with each block representing the average of 10 cabbages. Different letters denote statistical differences as determined by one-way ANOVA coupled with LSD post-hoc test (*p* < 0.05).

Three lines of male parents, QP03, DHP37, and QP15 (hereafter referred to as MP1, MP2, and MP3, respectively), three lines of female parents, QP13, QP04CMS, and QP16CMS (hereafter referred to as FP1, FP2, and FP3, respectively), and four of their F_1_ hybrids, HY1 (FP1 × MP1), HY2 (FP2 × MP1), HY3 (FP2 × MP2), and HY4 (FP3 × MP3) were studied (Figure 1a). The net weight of cabbage heads, polar head diameter, equatorial head diameter, weight of the distinguishable petioles, non-wrapper leaf weight, plant height, and plant diameter were evaluated and showed significant differences in the hybrids compared to their parents (Figure 1b–h). In addition, mid-parent heterosis (MPH) and high-parent heterosis (HPH)––the percentage deviation of the hybrid means (M_HY_) from the mean of the mid-parent (M_MiP_) and the high-parent (M_HiP_), respectively––were calculated. The MPH values were positive for all the traits in all the hybrids, which demonstrates improvements compared to the average of their parents. The HPH values, except for the non-wrapper leaf weight and plant height in HY1 and equatorial head diameter in HY3, were also positive, indicating improvements over the highest of the two parents. The increase in cabbage head weight was the most prominent, with MPH and HPH values ranging from 107–129% and 87–100%, respectively (Table 1), which suggests higher yield potentials of the hybrids.

### 2.2. Genetic Variations and Gene Expression Divergence between Hybrids and Their Parents

Transcriptome sequencing of the four cabbage hybrids and their six parental lines (each with three biological replicates) generated a total of 230.65 Gb of high-quality clean reads with a minimum of 6.28 Gb clean data for each replicate. A total of 771.2 million paired-end clean reads were sequenced with a Q30 score of ≥90.9%. The RNA-seq data was deposited to the NCBI sequence read archive (SRA) under the accession number PRJNA664256. After filtering out low quality reads, between 42.0 and 61.8 million paired-end reads were obtained for each replicate. Of these, about 67–70% of the paired reads aligned with the *B. oleracea* var. *capitata* reference genome and 66–68% of them aligned to unique positions. The number of reads aligned with positive and negative chains was almost identical. 

Genome-wide genetic variations play a substantial role in heterosis [10]. To determine the frequency of shared genetic variations, we examined the SNPs and InDels in the hybrids and parents. Compared to the reference genome, HY1–HY4 had 160,928, 177,148, 180,019, and 172,891 SNPs and 9112, 9617, 9743, and 9353 InDels, respectively. We observed that 3–12% of SNPs and 5–10% of InDels were unique to the hybrids. Also, 26–28% of SNPs and 14–15% of InDels were detected in the genomes of the hybrids and their two parents, with reference to the *B. oleracea* var. *capitata* reference genome. When the remaining SNPs and InDels were considered, the number of SNPs and InDels consistent with the female parent were slightly but consistently higher than that of the male parent, indicating a maternal parent bias in the transcriptome. This bias was particularly prominent in HY1, with 75,299 SNPs and 2359 InDels consistent with the female parent, compared to only 54,757 SNPs and 1642 InDels consistent with the male parent (Table 2). 

To evaluate the hybrid gene expression deviations from their parents, we performed pairwise expression comparisons and identified DEGs with more than two-fold expression difference and a false discovery rate of ≤0.01. Interestingly, all hybrids had a higher number of DEGs when compared with the male parent than when compared with the female parent (Table 3). This lower expression divergence between hybrids and female parents, together with the allelic expression bias towards female parents, suggests a closer genetic association between hybrids and female parents than hybrids and male parents.

In our hybrids, HY1 and HY2 shared the same male parent (MP1), and HY2 and HY3 shared the same female parent (FP2; Figure 1a). The lower gene expression divergence between hybrids and the female parent suggests that HY2 and HY3 may have a lower expression divergence compared to HY1 and HY2 (Table 3). However, there were 2342 DEGs between HY2 and HY3 compared to only 948 DEGs between HY1 and HY2 (Table 3). This discrepancy is likely due to the higher number of DEGs between MP1 and MP2 (5139) compared to that between FP1 and FP2 (2011) (Table 3). 

**Figure 2:**
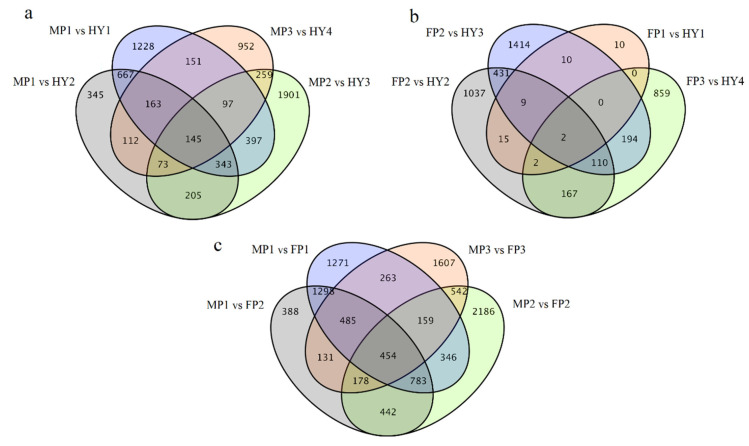
Venn diagram comparison of differentially expressed genes (DEGs) between hybrids and their parents. The common DEGs in different hybrids compared to their (**a**) male parent and (**b**) female parent, and (**c**) between female and male parent combinations used to generate the four different hybrids.

We further evaluated the DEGs between hybrids and parents as well as between male and female parents using Venn diagrams to identify common DEGs among hybrids (Figure 2). When hybrids and their male parents were compared, there were 145 DEGs common to all hybrids (Figure 2a). The comparison between hybrids and their female parents, however, revealed only two common DEGs (Figure 2b). This lower number of DEGs common to hybrids and their female parents can be due to the relatively smaller number of DEGs between hybrids and female parents; nevertheless, it also suggests a higher and steady divergence of hybrids from male parents. 

### 2.3. Functions of DEGs Associated with Cabbage Head Heterosis

To determine the roles of DEGs, we performed GO (Gene Ontology), COG (Clusters of Orthologous Groups), and KOG (Eukaryotic Orthologous Groups) analysis, as these databases cover detailed gene or protein function information. Among the four hybrids, HY3 had the highest number of DEGs compared to its parents. As the higher number of DEGs suggests stronger divergence of hybrids from their parents, GO, COG, and KOG pathway enrichment analysis between HY3 and its parents, MP2 and FP2 (MP2 vs. HY3 and FP2 vs. HY3), are described below; however, the evaluation of DEGs between other hybrids and their parents produced similar results.

In the GO enrichment analysis, “cellular processes” was the most overrepresented biological process subcategory, with 1479 and 2237 DEGs in HY3 compared to MP2 and FP2, respectively (the DEGs are given in the same order in all the following enrichment categories). “Single-organism process” (1411 and 2120) and “metabolic process” (1360 and 2053) were the other most represented subcategories. In the cellular component and molecular function categories, “cell part” (1738 and 2723) and “binding” (1060 and 1632) subcategories were the most enriched terms, respectively (Figure S1). In COG analysis, “transcription” (115 and 187), “signal transduction mechanisms” (117 and 164), and “replication, recombination, and repair” (107 and 161) were discovered as the top three enriched terms (Figure S2). The most enriched categories in KOG analysis were “posttranslational modification, protein turnover, chaperones” (102 and 191), “signal transduction mechanisms” (102 and 177), and “carbohydrate transport and metabolism” (94 and 127; Figure S2). 

To evaluate the transcription factors that may play a role in heterosis, we annotated the transcription factors represented by DEGs using the plant transcription factor database (PlantTFDB v4.0). A total of 54 MYB or MYB-related, LATERAL ORGAN BOUNDARIES (LOB) domain, and the KNOTTED1-LIKE HOMEOBOX (KNOX) transcription factors are among the differentially expressed genes that are likely involved in the growth and development of cabbage head leaves. The KNOX, MYB, and LOB domain transcription factors interact with each other, regulating the shoot morphogenesis and leaf patterning in the apical meristem [27,28]. The BELL-like homeobox and HOMEO-DOMAIN LEUCINE ZIPPER (HD-ZIP) transcription factors, which establish the polarity and leaf outgrowth [29], and the AUXIN RESPONSE FACTORs, which regulate the expression of auxin-responsive genes [30], were also abundant (Table S1). These diverse categories indicate the regulatory roles of extensive biosynthetic, metabolic, and signal transduction pathways behind heterosis.

### 2.4. Gene Expression Inheritance Patterns in the Cabbage Hybrids

**Figure 3:**
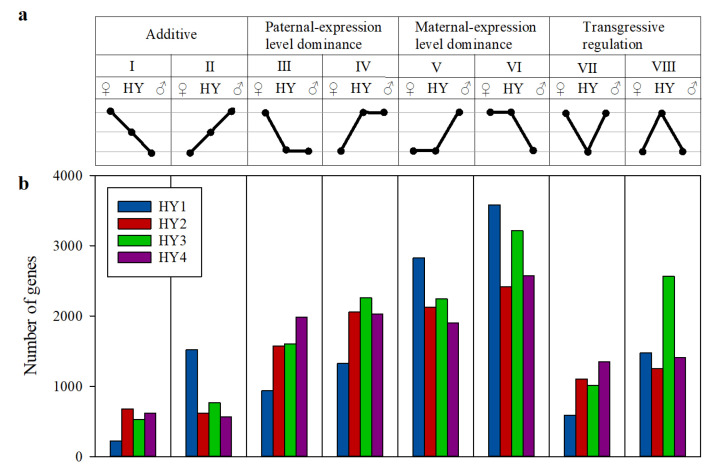
Gene expression inheritance patterns in the cabbage hybrids. (**a**) The eight different gene expression inheritance patterns based on the hybrid gene expression level compared to its parents: additive (I and II), paternal expression level dominance (low-parent dominance and high-parent dominance; III and IV), maternal expression level dominance (low-parent dominance and high-parent dominance; V and VI), transgressive regulation (upregulation and downregulation; VII and VIII). (**b**) The number of genes representing each expression inheritance category in the four hybrids.

To gain an overall insight into gene expression changes, we classified the inheritance patterns of DEGs as additive and non-additive. The non-additive inheritance was divided into paternal expression dominance, maternal expression dominance, and transgressive expressions. The transgressive category was further divided to differentiate up and down regulations, and the parental dominance categories were further divided to differentiate high-parent dominance and low-parent dominance. In total, there were eight different expression inheritance categories (I–VIII; Figure 3). Approximately 66–70% of the DEGs in the hybrids showed an expression level dominance, 9–14% showed an additive inheritance pattern, and 17–25% were transgressive. Among those exhibiting dominance, the number of genes showing high-parent dominance was higher than those showing low-parent dominance (Figure 3; compare category III with IV, and V with VI). Similarly, transgressive up-regulation was predominant over transgressive down-regulation (Figure 3; compare category VII with VIII). Of the genes showing expression level dominance, 53–74% showed maternal expression level dominance, whereas only 26–47% showed paternal expression level dominance, which again indicates a maternal bias in the expression inheritance (Figure 3; categories III–VI). 

**Figure 4:**
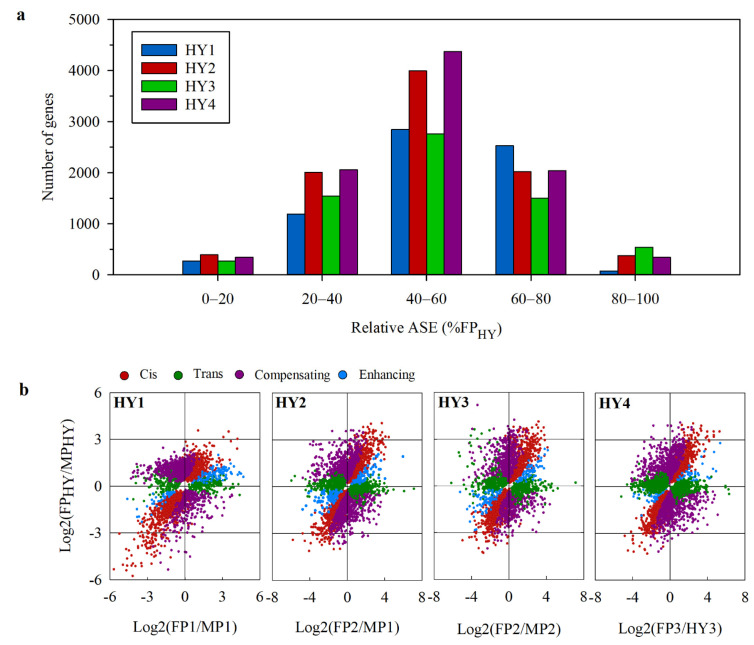
Relative allele-specific expression in the cabbage hybrids. (**a**) The number of genes representing differential expressions as grouped by the relative expression level of maternal alleles (% FP_HY_). Genes in the 40–60 category were considered to have a balanced allelic expression. Genes in the 60–80 and 80–100 categories show a female allele bias. Genes in the 0–20 and 20–40 categories show an expression bias toward the male allele. (**b**) The log2 expression ratios of maternal to paternal alleles in the parents vs. hybrids. Each point represents a single gene with colors representing the regulatory divergence category. MP_HY_ and FP_HY_: maternal and paternal allelic expression levels in the hybrids, respectively; MP and FP: maternal and paternal expression levels in the parents, respectively.

To further evaluate the maternal expression bias, we selected the genes with parent-specific SNPs and determined relative allele-specific expressions (ASEs), the percentage of female parent alleles in the transcriptome (% FP_HY_). In each hybrid, representation of female parent alleles in the transcriptome for approximately 41–48% of the genes was between 40–60% (40–60 category in Figure 4a), and therefore, these genes showed no clear expression bias [31]. For the remaining genes, HY1 and HY3 showed a maternal bias (60–100% category), but the other two hybrids showed no clear bias to either parent (Figure 4a). We further plotted the expression ratio of each allele in the parents (FP/MP) against their expression in the hybrids (FP_HY_/MP_HY_) on a logarithmic scale (Figure 4b). The distributions of alleles in these plots were mostly symmetrical and showed no clear bias to either parent. However, in HY1, more gene position in the upper quadrants indicates higher expressions of FP alleles (Figure 4b).

**Figure 5:**
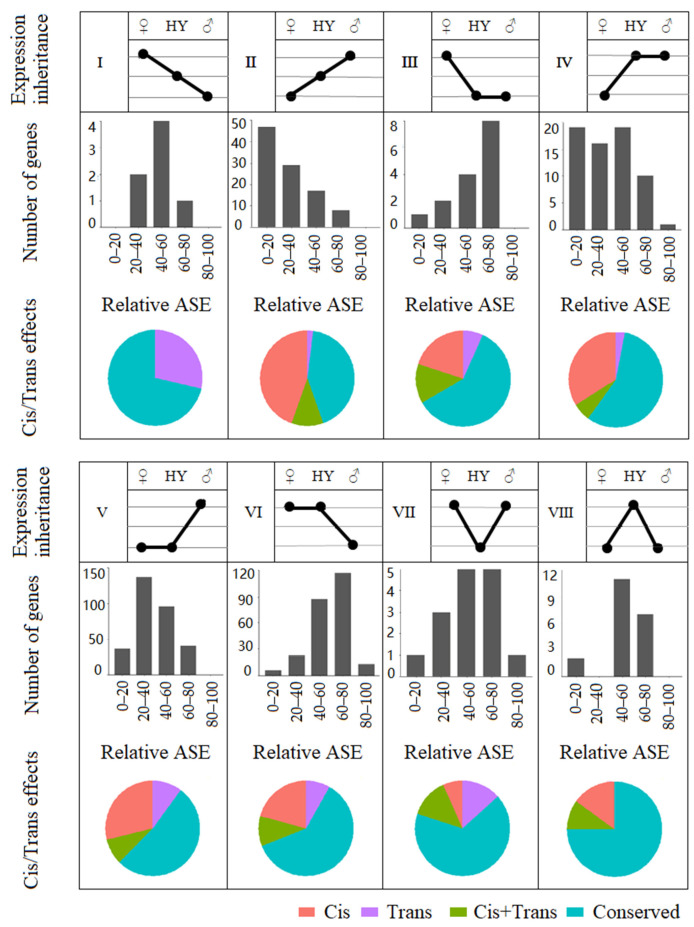
Comparison of relative allele-specific expressions (ASEs) and cis- and trans-effects according to seven expression inheritance patterns in HY1. The relative ASE represents the expression of maternal alleles as a percentage of the total gene expression in the hybrid (% FP_HY_). The pie charts show the proportion of genes showing cis-effects, trans-effects, or both cis- and trans- (cis + trans) effects. Genes showing no significant evidence of cis- or trans-effects were classified as conserved.

In all the hybrids, regardless of the expression inheritance patterns, the distribution ratio of relative ASEs was always unbalanced (Figure 5; see Figures S3–S5 for HY2–HY4). In the paternal expression dominance category showing high-parent dominance (category IV), relative ASE showed a paternal bias (0–40% category is predominant). In the maternal expression dominance category showing high-parent dominance (category VI), relative ASE also showed a maternal allele bias (60–100% category is predominant). In contrast, in the categories showing low-parent paternal and maternal dominance (categories III and V, respectively), relative ASE showed maternal and paternal allele bias, respectively. In the categories showing additive inheritance patterns, a maternal allele bias was observed in additive female parent > male parent conditions (category I), and a paternal allele bias was observed in additive male parent > female parent conditions (category II). The only exception was for HY1, where a paternal allele bias was observed in both situations. As for the transgressive up-regulation and down-regulation categories (categories VII and VIII), HY1 and HY2 showed a maternal allele bias, but no clear association could be seen in the other hybrids (Figure 5 and Figures S3–S5). The results suggest that relative ASE always shows a bias towards the parent showing the higher expression.

### 2.5. Cis- and Trans-Effects on Gene Expression Divergence

**Figure 6:**
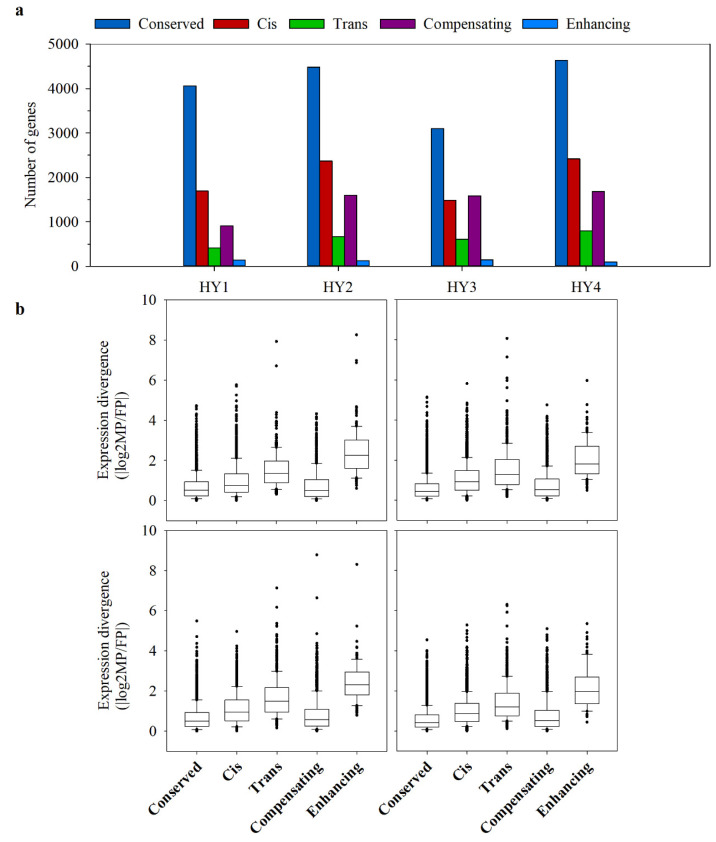
Cis- and trans-effects in the hybrids. (**a**) The number of genes in different regulatory categories. The ‘‘conserved” category represents the genes showing no clear cis- or trans-effects. Genes showing evidence of both cis- and trans-effects were subdivided as “compensating” and “enhancing”, where the cis- and trans-effects act in opposite directions and the same direction, respectively. (**b**) The absolute magnitude (fold-change) of parental expression divergence resulting from different regulatory effects. The median trans-effects were larger than the median cis-effects in all the four hybrids.

The gene expression divergence between hybrids and parents can result from both cis- and trans-regulatory changes [32]. To explore the genetic basis of expression divergence, we used parent-specific SNPs to compare ASE. After applying the quality control criteria, 31,962 (7223 genes), 52,177 (9246 genes), 34,653 (6937 genes), and 56,099 (9628 genes) SNPs with ≥10 read coverage were screened in HY1–HY4, respectively. Among them, 45–56% of the genes showed no expression divergence in the hybrids compared to their parents and were classified as conserved expressions (Figure 6a). The alleles that expressed differently between the parents and maintained that differential expression in the hybrids were classified as having only cis-effects. The alleles that expressed differentially in the parents but expressed equally in the hybrid were considered to have only trans-effects. In the remaining genes, cis- and trans-effects were co-acting (cis + trans effects) [11]. Cis-effects accounted for the majority of expression divergence in all the hybrids except HY3, where compensating cis + trans effects were predominant. Among the four hybrids, 21–26% of the genes analyzed showed cis effects, and 13–16% showed trans-effects (Figure 6a). The compensating cis + trans effects, where cis- and trans-effects act in the opposite directions, accounted for 13–23% of the ASE. In contrast, only 1–2% of the ASE was represented by enhancing cis + trans interactions, where cis- and trans-effects act in the same direction (Figure 6a). 

The contribution of cis- or trans-effects to the gene expression differences of the parents was inspected from the absolute magnitude of parental expression divergence in the hybrids. First, the median of both cis- and trans-regulations showed a high level of expression divergence relative to the conserved gene expression in all the hybrids, which indicates that both cis- and trans-regulations are behind parental expression divergence (Figure 6b). Second, although there are more transcripts with only cis-effects than with only trans-effects, the trans-effect appeared to contribute more to the gene expression divergence than the cis-effect (Figure 6b). 

### 2.6. The Relationship between Allelic Expression Regulation and Expression Inheritance in Hybrids

**Figure 7:**
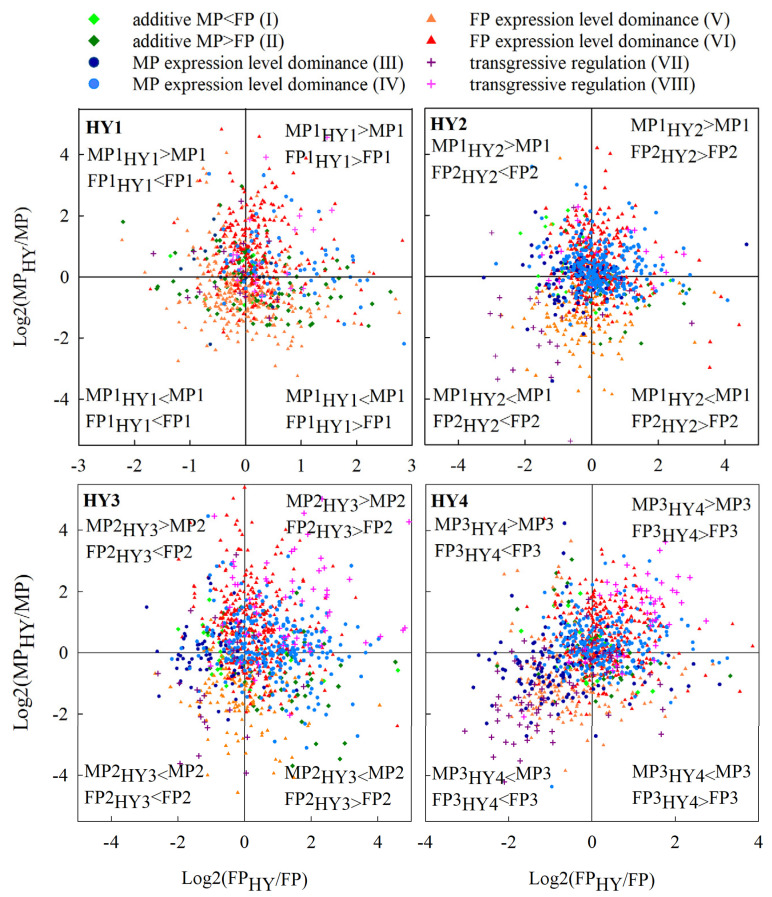
Allele-specific expression (ASE) variations of the differentially expressed genes (DEGs) in the hybrids. Each point in a plot represents a single gene. The position of each point represents the combined expression change of the two alleles in the hybrid with respect to the male (MP) and female (FP) parent.

Trans-regulatory factors commonly show asymmetric effects on the expression of parental alleles in hybrids, especially in allopolyploids [31]. For example, in F1 allotetraploids between *A. thaliana* and *A. arenosa*, the *A. arenosa* trans-factors tend to upregulate *A. thaliana* alleles, whereas *A. thaliana* trans-factors either upregulate or downregulate *A. arenosa* alleles [21]. To determine the roles of trans-regulatory factors in the parental expression bias of cabbage hybrids, we further assessed ASE variations in our hybrids. As the cis-regulatory factors in a parent genome and the hybrid genome representing that parent are similar, expression variations of alleles represent the effects of trans-regulatory factors [31]. To visualize the effects of trans-regulatory factors on the gene expression, we plotted the relative ASE of male parent alleles (MP_HY_/MP) against that of the female parent alleles (FP_HY_/FP) on a log2 scale [31]. The mostly symmetrical distribution of points in the plot shows that trans-regulatory factors from each parental genome can equally upregulate or downregulate alleles from the other genome, and the magnitude of expression variation in MP_HY_ alleles is similar to FP_HY_ alleles (Figure 7). Therefore, the FP and MP trans-regulatory factors appear to have a similar contribution to the hybrid gene expression divergence. The only exception was in HY1, where the expression variation of MP1_HY1_ alleles was higher than that of the FP1_HY1_ alleles, suggesting a higher impact of FP1 trans-regulatory factors on the MP1_HY1_ alleles compared to MP1 trans-regulatory factors on FP1_HY1_ alleles.

The distribution pattern of the genes in the scatter plot also shows that in the majority of genes classified as showing maternal expression level dominance with a high-parent dominance (category VI in Figure 3), the MP_HY_ alleles were mainly upregulated by FP trans-regulatory factors. In the genes showing a maternal expression level dominance with a low-parent dominance (category V), the MP_HY_ alleles were mostly downregulated by FP trans-regulatory factors. The same trend was observed in the paternal dominance. In the genes showing paternal expression level dominance with a high-parent dominance (category IV), the FP_HY_ alleles were mainly upregulated by MP trans-regulatory factors. In the genes showing a paternal expression level dominance with a low-parent dominance (category III in Figure 3), the FP_HY_ alleles were mostly downregulated by MP trans-regulatory factors. For the additive and transgressive categories, gene expression appears to be a combined effect of both male parent and female parent trans-regulatory factors (Figure 7). Overall, the expression level dominance towards a parent can be explained by the effect of that parent’s trans-regulatory factors on the other parental genome.

## 3. Discussion

### 3.1. Gene Expression Divergence between Cabbage Hybrids and Their Parents

In this study, we used transcriptome sequencing to evaluate the genomic and transcriptomic level changes underlying cabbage head heterosis. Many transcriptomic analyses in plants and other organisms show that gene expression in hybrids can be biased towards a particular parent [14,20,31,33,34,35]. In cabbage hybrids, the higher number of SNPs and InDels consistent with the female parent than with the male parent, suggests a closer genetic relationship between the female parent and the hybrids. The number of DEGs showing expression profiles similar to the female parent was also higher compared to male parents in all the hybrids. Thus, we deduced that the female parent likely plays a larger role than the male parent in the hybrid gene expression divergence. However, the present analysis was limited to a single stage of cabbage head development, and the gene expression bias may shift depending on the developmental stage. For example, in rice (*Oryza sativa* L. ssp. *indica*), transcriptome profiles of leaves were closer to the maternal parent at the early plant development, but closer to the paternal parent at later stages [34]. Also, without reciprocal crosses, we cannot rule out other possible factors that may cause the observed female parent bias. The parent-of-origin effects, also known as transgenerational effects, are associated with the parental genotype and can be influenced by the environmental conditions or physiological state. For example, the maternal genotype of the endosperm affects seed development, which may influence early seedling development and thereby plant vigor at later stages [36]. The parent-of-origin effects may also arise due to genomic imprinting, an epigenetic regulatory mechanism that causes one parental allele to be expressed prominently. In plants, genomic imprinting is usually limited to multiple genetic loci with single genes, and imprinted genes are almost entirely confined to endosperm. Therefore, genomic imprinting generally affects reproductive development [37]; however, we cannot exclude the possibility that this may cause long-term effects that appear in plants at later stages.

The other key questions here are whether the gene expression bias towards a particular parent has a positive or negative correlation with heterosis, and whether it is a cause or consequence of heterosis. Even though the answers to these questions are not clear, in rice, analysis of some selected loci suggests a negative correlation between hybrid yield and paternal gene expression bias [35]. In maize (*Zea mays* L.), a smaller positive relationship was reported between the yield and maternal expression bias [38]. Even so, whether these associations are responsible for heterosis or are merely a consequence of the phenotypes remains unknown. 

The high HPH and MPH values show that there is a significant improvement in the head weight and petiole weight of all the hybrids (Table 1). Many studies show that yield or biomass heterosis correlates with an increased level of metabolic activity [12,34,39]. In maize yield heterosis, for example, DEGs have been found to be significantly enriched in carbohydrate metabolism associated genes [40]. At the heading stage of rice, the DEGs mapped to quantitative trait loci (QTLs) for yield were also linked to carbohydrate metabolism [41]. We also observed a significant enrichment of carbohydrate transport and metabolism genes in the COG analysis of DEGs, which suggests a correlation in biomass heterosis and carbohydrate metabolism in cabbage hybrids.

In the GO function, COG, and KOG pathway enrichment analyses, metabolic process, catalytic activity, replication, recombination and repair, transcription, and signal transduction mechanisms were among the most overrepresented terms (Figures S1 and S2). Many of these terms are commonly represented in the heterosis of various other plant species. For example, evaluation of DEGs associated with rice seedling heterosis has shown that transcription, metabolism of cofactors and vitamins, amino acid metabolism, and biosynthesis pathways of secondary metabolites are significantly enriched [42]. Genome-wide comparisons of maize hybrids indicated that biological processes, including metabolism, signal transduction, transport, biological regulation, and development are the main functions of the genes represented by DEGs [43]. In Arabidopsis, pathways contributing to growth heterosis were also composed of enrichment categories similar to those observed in cabbage [44]. Altogether, the similarity of enriched pathways associated with different growth-related traits in diverse plant species suggests that those traits are mostly regulated by broader but relatively similar pathways rather than a single gene or locus affecting a specific pathway [45].

### 3.2. Maternal Expression Level Dominance is Predominant in Cabbage Hybrids

Both additive and non-additive gene expression inheritance patterns contribute to the gene expression divergence in hybrids; however, the relative contribution of each inheritance pattern to heterosis is not clear. In our cabbage hybrids, more than 86% of genes showed non-additive expression inheritance patterns. This high percentage indicates that the expression divergence of most hybrid genes is not merely a result of the combined effect of allelic expressions, but a consequence of diverse gene expression regulatory mechanisms. A number of recent studies, including in maize [46], soybeans [47], rubber trees [10], interspecific hybrids between *Brassica napus* and *B. rapa* [48], and cauliflower [49], show a prevalence of non-additive gene action in the hybrids. Non-additive gene expressions may have both direct and indirect contributions to heterosis. For instance, in *Arabidopsis* hybrids, non-additive genes likely enhance metabolic activities, which leads to improved resource utilization and increased seedling growth rates [50]. In soybeans, 19 non-additive genes associated with nitrogen use efficiency likely play a role in heterosis by improving the protein content of seeds [47]. 

The expression of a given gene in a hybrid can be non-additive when it is showing a maternal or paternal expression dominance. Also, the expression of some genes can be independent of the parental expression levels (transgressive regulation). Of the genes showing non-additive expressions in our hybrids, 72–81% (66–70% of the total genes) showed dominant expression patterns. Approximately 53–74% of these genes showed maternal expression level dominance, and only 26–47% showed paternal expression level dominance. Therefore, it is likely that cabbage hybrids predominantly show an expression level dominance with a maternal bias when establishing heterosis. This observation is consistent with various other studies, where parental expression dominance has been shown to predominate in the hybrids [15,31,48,51]. 

### 3.3. Cis-Regulation Predominates the Hybrid Gene Expression Divergence

Gene expression is controlled by a complex regulatory network, which includes interactions between DNA, RNA, proteins, and environmental factors. The quantitative changes in gene expressions, however, are directly regulated by the cis- and trans-effects [32]. If differences of allelic regulation are only due to cis-regulatory changes, the expression of the allele in hybrids can be expected to be additive. However, hybridization exposes the parental alleles to trans-regulatory factors originating from both parents, and therefore, the differences in allelic regulation may also be due to trans-effects. Numerous studies have shown that non-additive gene expression mainly results from the trans-effects, and may cause the gene expression in the hybrids to deviate significantly from additive expression [31,46,51,52]. In cabbage hybrids, of the genes showing non-conserved expressions, 39–54% showed cis-effects, compared to only 13–16% showing trans- effects (Figure 6a). However, in the parents, the majority of gene expression differences were regulated by trans-regulatory factors (Figure 6b).

Plants have to adapt to the changing environment continually. Enhancing cis + trans interactions increase gene expression divergence and promote disruptive or diversifying plant characteristics. In contrast, compensating cis + trans interactions reduce gene expression divergence (stabilizing selection), since the cis effects are compensated by opposite actions of trans-effects or vice versa. [21]. Stabilizing selection tends to keep the stability of internal cellular functions by maintaining the expression of genes involved in metabolic and biosynthetic processes at balanced levels [53]. Among the genes showing non-conserved expressions, 29–41% showed compensating cis + trans interactions, compared to only 2–4% showing enhancing cis + trans interactions (Figure 6a). Therefore, stabilizing selection effects appear to be common in maintaining the gene expression levels in cabbage hybrids. Altogether, it is likely that a complex combination of cis- and trans- effects determines the gene expression inheritance patterns.

Since trans-effects increased with the parental expression divergence, we further assessed the role of trans-effects in modulating allelic expression and gene expression inheritance patterns. The mostly symmetrical distribution of alleles in Figure 7 suggests that the effects of trans-regulatory factors from the two parents are generally balanced. The regulation of non-dominant parent alleles by the trans-regulatory factors of the dominant parent, without a bias towards a parent, likely caused the majority of the expression level dominance. The other inheritance patterns are likely determined by the combined effect of trans-regulatory factors from both parents acting on each other’s alleles (Figure 7). These observations are consistent with many studies, including in allopolyploid cotton [13], *Cirsium arvense* [20], and fungal allopolyploids [54], where trans-regulatory factors play a prominent role in the regulation of hybrid expression inheritance patterns. For example, in the interspecific hybrids of *Coffea canephora* and *C. eugenioides*, allelic expression patterns, including additive and transgressive categories, have shown to depend on the combined effect of trans-regulatory factors from the parental genomes, while asymmetric effects of trans-regulatory factors appear to cause biased expression level dominance [31].

In conclusion, our data suggest that genetic divergence between hybrids and parents, cis- and trans-effects, and the gene expression patterns play important roles in establishing cabbage heterosis. The larger number of SNPs and InDels consistent with the female parent and the expression bias represented by DEGs indicate that the female parent has a higher contribution to the gene expression divergence. The expression inheritance patterns suggest a female parent expression level dominance and a mostly non-additive expression of hybrid genes. Both cis- and trans-effects tend to mediate gene expression divergence in the hybrids, but with a comparatively higher contribution from the cis-effects.

## 4. Materials and Methods 

### 4.1. Plant Materials and Measurement of Horticultural Traits

Cabbage (*Brassica oleracea* L. var. *capitata*) seeds were planted in a research field at the Northwest A&F University, Shanxi, China, under normal farming conditions in late July of 2017. The female parents FP2 and FP3 belonged to the Ogura cytoplasmic male sterile (CMS) line and were obtained via seven generations of backcrossing [55]. The remaining female parent (FP1) was a new and stable cabbage variety that had been self-pollinated for six generations. The three male parents (MP1, MP2, and MP3) were double haploid lines obtained by microspore culture. These lines were selected as parents due to their high crack resistance, high yield, consistency of quality traits, and our observations that their hybrids showed superior head trait phenotypes as described in the results section. Some noticeable phenotypic differences of these lines are described in Table S2. 

Plants were grown in randomized blocks with three replications per line and 20–30 plants per replication. The cabbage heads were harvested when 80% of the head leaves were at commercial maturity. In each replicate, the largest and the smallest cabbages were removed to gain a representative sample of average-sized cabbage heads. The following seven horticultural traits were evaluated: net head weight, polar head diameter, equatorial head diameter, the total weight of the distinguishable petioles (main petioles), non-wrapper leaf (photosynthetic outer leaves that are not part of the cabbage head) weight, plant height, and plant diameter (Figure S6). 

MPH and HPH were calculated using the equations below [56]. Statistical significance was determined by one-way ANOVA with LSD post-hoc test (SPSS, Ver.16.0; *p* < 0.05).

MPH (%) = (M_HY_ − M_MiP_)/M_MiP_ × 100 (1)

HPH (%) = (M_HY_ − M_HiP_)/M_HiP_×100 (2)

### 4.2. RNA Extraction and Sequencing

The outer layer of cabbage head leaves (first layer of wrapper leaves) was used for RNA extraction when 80% of the head leaves were at commercial maturity. Approximately 10 cm^2^ of the wrapper leaf was collected from the top center region, frozen immediately in liquid nitrogen, and stored at –80 °C until RNA extraction. Each sample was composed of leaves from five randomly selected cabbages. There were three biological replicates for each line. Total RNA was extracted from finely ground leaves using a TRIzol-based method (Tiangen, Beijing, China). RNA concentration and integrity were assessed on an Agilent Bioanalyzer 2100 system using the RNA Nano 6000 Assay Kit (Agilent Technologies, Chandler, USA). The RNA integrity number (RIN) was 8.1 or higher for all samples. 

Sequencing libraries were generated using NEBNext Ultra RNA Library Prep Kit for Illumina (NEB, Ipswich, MA, USA), and index codes were added to match sequences to each sample. Briefly, mRNA was purified from total RNA using poly-T oligo-attached magnetic beads. Fragmentation was carried out using divalent cations under elevated temperature in NEBNext First-Strand Synthesis Reaction Buffer. The first-strand cDNA, synthesized using random hexamer primers and M-MuLV reverse transcriptase, was treated with RNase H and used for second-strand cDNA synthesis with DNA Polymerase I. The overhangs were converted into blunt ends by exonuclease. After the adenylation of 3′ ends, the DNA fragments were prepared for hybridization by ligating NEBNext adaptors bearing hairpin loop structures. The library fragments were purified with AMPure XP system (Beckman Coulter, Indianapolis, Indiana, USA) to select cDNA fragments of approximately 240 bp in length. Then, PCR was performed with Phusion High-Fidelity DNA polymerase, universal PCR primers, and Index (X) Primer. PCR products were purified (AMPure XP system), and library quality was assessed on an Agilent Bioanalyzer 2100 system. Finally, clustering of the index-coded samples was performed on a cBot Cluster Generation System, using a TruSeq PE Cluster Kit v4-cBot-HS (Illumina). Sequencing was done in an Illumina HiSeq Xten platform, and paired-end reads were generated.

### 4.3. Quality Control and Read Mapping

The raw data in FASTQ format were processed using in-house Perl scripts. Clean data (clean reads) were obtained by removing reads containing adapter, reads containing ploy-N, and low-quality reads from raw data. The Q20 and Q30 values, GC content, and the extent of sequence duplication level in the filtered data were determined. Thus, all downstream analyses were based on high-quality clean data. When the clean reads were mapped to the *B. oleracea* var. *capitata* reference genome (RefGen_v2.1) [57], only the reads with a perfect match or one mismatch were further analyzed and annotated. Tophat (v 2.1.1) was used to map the clean reads to the reference genome [58]. Transcriptome sequencing can be simulated as a process of random sampling, that is, to randomly extract sequence fragments from any nucleic acid sequence in a sample transcriptome. The number of fragments extracted from a gene (or transcript) obeys Beta Negative Binomial Distribution [59]. Based on this mathematical model, the Cuffquant and Cuffnorm components of the Cufflinks software were used to quantify the expression levels and genes through the location information of mapped reads on genes [58]. The expression level of each gene was calculated and normalized by the fragments per kilobase of transcript per million mapped reads (FPKM) [60]. 

### 4.4. Validation of RNA-Seq Data by qRT-PCR

To verify the accuracy of the RNA-seq data, we randomly selected ten genes from the DEGs and performed quantitative reverse transcription PCR (qRT-PCR; Table S3). The peptidyl-prolyl cis-trans isomerase (cyclophilin) gene was used as the internal reference [61]. First-strand reverse transcription was conducted using the PrimeScript RT reagent kit with gDNA Eraser (Perfect Real Time; Takara Bio., Shiga, Japan). The qRT-PCR reactions were performed with the EvaGreen 2X qPCR MasterMix Kit in an ABI 7500 Quantitative PCR System (Applied Biosystems, Foster City, CA, USA) with four technical replicates and ten samples. The cycle parameters were 95 °C for 10 min, followed by 35 amplification cycles at 60 °C for 1 min and 94 °C for 15 s. Results were analyzed using the 2^-ΔΔCt^ method [62]. The expression trends seen in the qPCR data were similar to that of the RNA-seq data, which confirms the reliability of the RNA-seq results (Table S4). 

### 4.5. Analysis of the SNP Sites and the DEGs

Picard-tools (v 1.41) and SAMtools (v 0.1.18) were used to sort and remove duplicated reads, and then the bam alignment results of each sample were reordered [63]. GATK2 software was used to perform SNP and InDel analysis [64]. GATK standard filter method was used (cluster window size: 10; MQ0 ≥ 4; MQ0/(1.0*DP) > 0.1; QUAL < 10; QUAL < 30.0, QD < 5.0 or HRun > 5) to filter raw files, and SNPs with a set value > 5 were retained.

The DEGs among each comparison group were analyzed using the R package DESeq (v 1.10.1) [65]. The resulting P values were adjusted using the Benjamini and Hochberg’s approach to control the false discovery rate. Genes with an adjusted *p*-value of ≤ 0.01 and fold change of ≥2 were considered as differentially expressed. GO enrichment analysis of the DEGs was implemented by the Goseq R package based on Wallenius’ noncentral hypergeometric distribution. The enriched GO terms were adjusted by multiple testing (*p*-value < 0.05) [66]. 

KOBAS software was used to analyze the enrichment of DEGs in KEGG pathways [66]. Pathways with an adjusted *p*-value of ≤ 0.05 were considered as significantly enriched. The BLAST homologous sequence analysis tool and the plant transcription factor database (v4.0; http://planttfdb.cbi.pku.edu.cn/) were used to annotate the transcription factors associated with the growth and development of cabbage head leaves. 

### 4.6. Inheritance Classification and Cis- and Trans-Regulatory Effects

Inheritance classification was performed as described in [31,33]. The DESeq package was used to normalize the expression value of parents and their hybrids. Expression inheritance was determined by subtracting the log-transformed expression values of each parent from those of the hybrids. The hybrid genes showing a total expression deviation of more than 1.25-fold from either parent were considered to have a non-conserved inheritance. Gene expressions in the hybrids that were lower than one parent but higher than the other parent were considered as showing mid-parent levels and were classified as additive. Genes showing expression levels similar to one parent were classified as dominant (maternal or paternal and high-parent or low-parent dominance). Hybrid gene expressions greater or less than both parents were classified as showing transgressive-up regulations and transgressive-down regulations, respectively [31,33]. 

To infer hybrid ASE levels, parent-specific SNPs were identified using custom Perl scripts. Only the divergent polymorphic nucleotide sites where accessions of both parents were homozygous for a given difference were retained. SNPs with a minimum of 10x read coverage in the hybrids were used to determine allele-specific expressions (ASEs) and to distinguish paternal and maternal alleles in the hybrids. For the quantification of ASE, the DESeq package was used to normalize mapped read depth coverage at SNP sites in the hybrid and parental alignments. After applying quality control criteria, 31,962, 52,177, 34,653, and 56,099 SNPs and 7223, 9246, 6937, and 9628 genes with ≥10 read coverage were identified in HY1–HY4, respectively. Relative ASE was calculated as the percentage of allele-specific read counts representing the female parent (Fisher’s exact test, *p*-value < 0.05) [31]. 

The cis- and trans-regulatory effects on the gene expression of hybrids were determined using ASE. Since the parental alleles in a hybrid are in the same cellular environment and share the same trans-regulatory factors, there is no trans-regulatory effect on the expression differences between parental alleles; therefore, the ASE divergence in the hybrids reflects the cis-effect. The trans-effect could be estimated by subtracting the cis-effect from the overall expression divergence between male and female parents [21,32]. The cis-effects were determined by evaluating the ASE ratios in a given hybrid (two-sided prop. test in R, H_0_: FP_HY_/MP_HY_ = 1, Benjamini–Hochberg method). The trans-effects were determined by comparing parental expression ratio with the allelic expression ratio of a given hybrid (H_0_: FP/MP= FP_HY_/MP_HY_, Benjamini–Hochberg method).

## Figures and Tables

**Table 1:** Mid-parent heterosis (MPH) and high-parent heterosis (HPH) values for different horticultural traits of the cabbage hybrids.

Hybrid	Net Head- Weight	Weight of the Distinguishable Petioles	Polar Head Diameter	Equatorial Head Diameter	Non- Wrapper Leaf Weight	Plant Height	Plant Diameter
**MPH (%)** **^╪^**							
**HY1**	121.9 ± 2.2	71.7 ± 4.9	25.9 ± 0.6	27.9 ± 0.7	27.3 ± 1.0	7.1 ± 1.4	10.2 ± 0.7
**HY2**	129.2 ± 4.4	72.6 ± 6.8	18.9 ± 0.6	27.4 ± 0.7	32.4 ± 2.7	15.1 ± 1.3	30.2 ± 0.2
**HY3**	106.7 ± 3.5	27.1 ± 4.8	11.8 ± 0.7	5.0 ± 0.8	23.3 ± 2.3	10.4 ± 1.8	19.8 ± 0.3
**HY4**	122.6 ± 4.1	77.4 ± 10.0	9.1 ± 0.2	30.3 ± 2.2	60.5 ± 4.0	27.5 ± 0.6	32.3 ± 0.6
**HPH (%)**							
**HY1**	97.5 ± 2.3	42.6 ± 2.0	18.6 ± 0.7	25.1 ± 1.3	−5.9 ± 0.2	−11.3 ± 1.3	2.3 ± 0.3
**HY2**	94.5 ± 3.2	72.1 ± 6.8	5.4 ± 0.7	13.2 ± 0.6	21.7 ± 2.4	12.3 ± 1.8	25.5 ± 0.5
**HY3**	86.7 ± 3.1	16.1 ± 3.9	7.1 ± 0.3	−0.9 ± 1.1	19.6 ± 2.2	7.9 ± 5.1	5.3 ± 0.8
**HY4**	99.9 ± 3.2	45.9 ± 6.2	3.1 ± 0.3	23.3 ± 2.1	23.1 ± 2.3	15.8 ± 0.5	21.7 ± 0.9

^╪^ The percentage deviation of the hybrid means from the mean of the mid-parent and the high-parent. Data are means ± SD (n = 3 blocks, with each block representing the average of 10 cabbages).

**Table 2:** The genetic variations between cabbage hybrids and their parents.

Genetic Variation	Hybrid			
	HY1	HY2	HY3	HY4
**SNPs**				
Number of hybrid specific SNPs	5180	11,057	22,290	9698
Number of SNPs consistent with both parents	44,795	47,246	46,570	45,092
Number of SNPs consistent with the male parent	54,757	72,233	62,763	72,271
Number of SNPs consistent with the female parent	75,299	77,013	76,463	72,426
**InDels**				
Number of hybrid specific InDels	457	677	921	591
Number of InDels consistent with both parents	1309	1415	1433	1380
Number of InDels consistent with the male parent	1642	2124	2007	2189
Number of InDels consistent with the female parent	2359	2428	2398	2258

**Table 3:** Comparisons of DEGs between hybrids and parents, or among hybrids or parents.

Comparison Group	Number of DEGs	Up-Regulated	Down-Regulated
**FP1 × MP1** **→** **HY1**			
**FP1 vs. HY1**	48	25	23
**MP1 vs. HY1**	3191	1562	1629
**FP2 × MP1** **→** **HY2**			
**FP2 vs. HY2**	1773	1142	631
**MP1 vs. HY2**	2053	1272	781
**FP2 × MP2** **→** **HY3**			
**FP2 vs. HY3**	2170	1454	716
**MP2 vs. HY3**	3420	2098	1322
**FP3 × MP3** **→** **HY4**			
**FP3 vs. HY4**	1334	874	460
**MP3 vs. HY4**	1952	1118	834
**Hybrids/parents**			
**HY1 vs. HY2**	948	523	425
**HY1 vs. HY3**	1294	870	424
**HY1 vs. HY4**	1991	1082	909
**HY2 vs. HY3**	2342	1293	1049
**HY2 vs. HY4**	2872	1208	1664
**HY3 vs. HY4**	2475	1053	1422
**MP1 vs. MP2**	5139	2369	2770
**MP1 vs. MP3**	4391	2047	2344
**MP2 vs. MP3**	4915	2503	2412
**FP1 vs. FP2**	2011	1132	879
**FP1 vs. FP3**	3683	1933	1750
**FP2 vs. FP3**	3699	1792	1907

## Data Availability

The sequencing reads generated in this study were deposited in the NCBI Sequence Read Archive (SRA) under accession number PRJNA664256.

## Supplementary Materials

The following are available online at https://www.mdpi.com/2223-7747/10/2/275/s1. Figure S1. GO pathway enrichment analysis for DEGs of FP2 vs. HY3 and MP2 vs. HY3. The dark color bars show percentages/numbers of DEGs representing each category. The light color bars show the percentage/numbers of the GO categories of all the expressed genes detected. Figure S2. COG and KOG pathway enrichment analysis of DEGs for FP2 vs. HY3 and MP2 vs. HY3. (A,B) COG pathway enrichment analysis of DEGs for FP2 vs. HY3 and MP2 vs. HY3, respectively. (C,D) KOG pathway enrichment analysis of DEGs for FP2 vs. HY3 and MP2 vs. HY3, respectively. Figures S3–S5. Relative allele-specific expressions (ASEs) and cis- and trans-effects of HY2, HY3, and HY4, respectively. The relative ASE represents the expression of maternal alleles as a percentage of total gene expression in the hybrid (%FPHY). The pie chart shows the proportion of genes showing cis-effects, trans-effects, or both cis- and trans- (cis + trans) effects. Genes showing no significant evidence of cis- or trans- effects were classified as conserved. Figure S6. Horticultural traits of the cabbage head. The non-wrapper leaves represent the photosynthetic outer leaves that are not part of the cabbage head. The distinguishable petioles of each layer were cut out to assess the total weight of the main petioles (shown in white arrows). Table S1. Selected transcription factors represented by DEGs that are likely involved in the growth and development of cabbage heads. Table S2. Backgrounds of the six parental inbred lines. Table S3. List of the forward (F) and reverse (R) primer combinations used in the qRT-PCR analysis. Table S4. Comparison of qRT-PCR and RNA-Seq expression data.
