# Supplementary material for: Comparative Transcriptomic Analysis of Gene Expression Inheritance Patterns Associated with Cabbage Head Heterosis
Source: Plants (Basel). 2021 Jan 31;10(2):275. doi: 10.3390/plants10020275 (PMC7912167; doi:10.3390/plants10020275)
Supplement: Supplementary file 1 [file plants-10-00275-s001.zip › Supplementary materials/Table S2.docx]

**Table S2.** Backgrounds of the six parental inbred lines.

| **Name** | **True Name** | **General growth period** | **Non-wrapper leaf color** | **Wax content on leaf surface** | **Cabbage head shape** | **Head compactness index** |
| --- | --- | --- | --- | --- | --- | --- |
| **FP1** | QP13 | 65 d | Gray green | High | Round | 0.62 |
| **FP2** | QP04CMS | 80 d | Gray green | High | Round | 0.59 |
| **FP3** | QP16CMS | 65 d | Gray green | High | Flat round | 0.62 |
| **MP1** | QP03 | 70 d | Green | Low | Round | 0.68 |
| **MP2** | DHP37 | 60 d | Green | Low | Round | 0.57 |
| **MP3** | QP15 | 90 d | Yellow green | Low | Flat round | 0.56 |
