# Supplementary material for: Comparative Transcriptomic Analysis of Gene Expression Inheritance Patterns Associated with Cabbage Head Heterosis
Source: Plants (Basel). 2021 Jan 31;10(2):275. doi: 10.3390/plants10020275 (PMC7912167; doi:10.3390/plants10020275)
Supplement: Supplementary file 1 [file plants-10-00275-s001.zip › Supplementary materials/Table S3.docx]

**Table S3.** List of the forward (F) and reverse (R) primer combinations used in the qRT-PCR analysis

| **Gene name** | **Primers (5’-3’)** |
| --- | --- |
| *CYP* | F:AGGAGGAGATTTCACCGC; R: TCTCTAACGACATCCATCCC |
| Bo6g011000 | F: AGCTTGGTCATGGCTCTAAC; R: CTGAGTTGGACTGTGAAGGATAA |
| Bo9g010160 | F: GGAGCAGCAAGAGGCTATAAA; R: GAGATGAAGCTCTGCACCTAAT |
| Bo5g088280 | F: CCTACTTGCACTCCGACTAATG; R: ACGCCCTTGTGTCTTTCTATC |
| Bo5g149860 | F: GAGGTTGATACAAGAGGCAAAGA; R: TCCAGCTGCCACAAACATAG |
| Bo6g118330 | F: GGAGTGGAGAGACAGAGGATAA; R: ACGGAGAAGCTGGTTGTATTT |
| Bo5g152690 | F: CATCAAGACCCTCTCTGCTTATT; R: TTCTCTCCTCTCGGTGTTCT |
| Bo5g130530 | F: CAACATTCTGGTGATGTGTGATC; R: AACTTTGGAGCTGCTGAAGA |
| Bo3g057170 | F: ACCTCGAGAAGGGAGGTATT; R: CCCAGTCCAAGTAGAAAGAGTG |
| Bo1g039360 | F: TCAGAGCTGGTGCCATTTAC; R: CAACCTCAGCTGCTACATCTAC |
| Bo1g084830 | F: TTTGCGGGAGAGACATTTAGG; R: GTCAGTGCGGTTAGATGAGAAG |
