# Supplementary material for: Comparative Transcriptomic Analysis of Gene Expression Inheritance Patterns Associated with Cabbage Head Heterosis
Source: Plants (Basel). 2021 Jan 31;10(2):275. doi: 10.3390/plants10020275 (PMC7912167; doi:10.3390/plants10020275)
Supplement: Supplementary file 1 [file plants-10-00275-s001.zip › Supplementary materials/Table S4.docx]

**Table S4.** Comparison of qRT-PCR and RNA-Seq expression data.

| **GENE** | **FP1 vs HY1** | |  | **FP2 vs HY2** | |  | **FP2 vs HY3** | |  | **FP3 vs HY4** | |
| --- | --- | --- | --- | --- | --- | --- | --- | --- | --- | --- | --- |
|  | **qPCR** | **RNA-seq** (log2 ratio) |  | **qPCR** | **RNA-seq** (log2 ratio) |  | **qPCR** | **RNA-seq** (log2 ratio) |  | **qPCR** | **RNA-seq** (log2 ratio) |
|  | log2 ratio |  |  | log2 ratio |  |  | log2 ratio |  |  | log2 ratio |  |
| Bo6g011000 | -0.89 | -infinity |  | -1.84 | -1.55 |  | -0.45 | -2 |  | 0.16 | 0.08 |
| Bo9g010160 | -0.64 | -0.09 |  | -1.69 | -1.4 |  | -0.85 | -1.23 |  | -1.12 | -infinity |
| Bo5g088280 | 0.61 | 1.16 |  | 1.21 | 1.47 |  | 1.33 | 1.96 |  | -2.47 | -infinity |
| Bo5g149860 | 0.9 | 0.56 |  | 2.81 | 1.58 |  | 3.33 | 2.74 |  | -2.64 | -0.05 |
| Bo6g118330 | 6.3 | 0.89 |  | 0.32 | 1.93 |  | 0.89 | 1.1 |  | -6.64 | -0.67 |
| Bo5g152690 | 6.5 | 1.08 |  | 8.28 | 1.97 |  | 3.18 | 1.96 |  | 0.88 | 0.05 |
| Bo5g130530 | -0.45 | -infinity |  | 2.06 | 2.5 |  | 1.47 | 2.57 |  | 0.68 | 0.16 |
| Bo3g057170 | 2.15 | 2.26 |  | 3 | 2.54 |  | 2.52 | 2.28 |  | -2.94 | -0.19 |
| Bo1g039360 | 0.28 | 2.24 |  | 1.53 | 2.67 |  | 2.03 | 2.85 |  | -3.18 | -1.07 |
| Bo1g084830 | 0.46 | 0.08 |  | 8.47 | 9.6 |  | 10.6 | 9.55 |  | -4.32 | -1.02 |
