# Supplementary figures and images for: Comparative Transcriptomic Analysis of Gene Expression Inheritance Patterns Associated with Cabbage Head Heterosis
Source: Plants (Basel). 2021 Jan 31;10(2):275. doi: 10.3390/plants10020275 (PMC7912167; doi:10.3390/plants10020275)

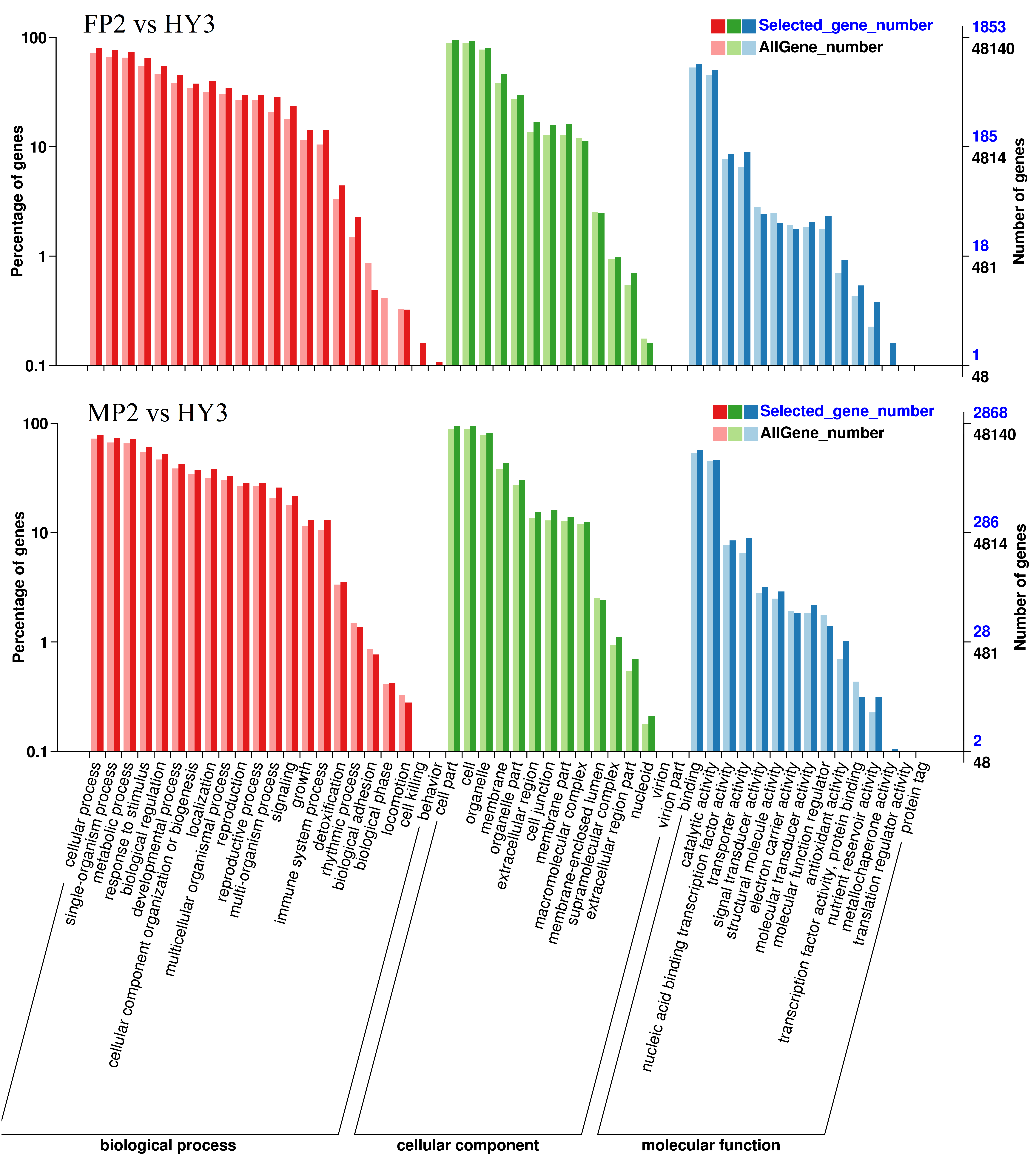

Supplement: Supplementary file 1 [file plants-10-00275-s001.zip › Supplementary materials/Figure S1.tif]

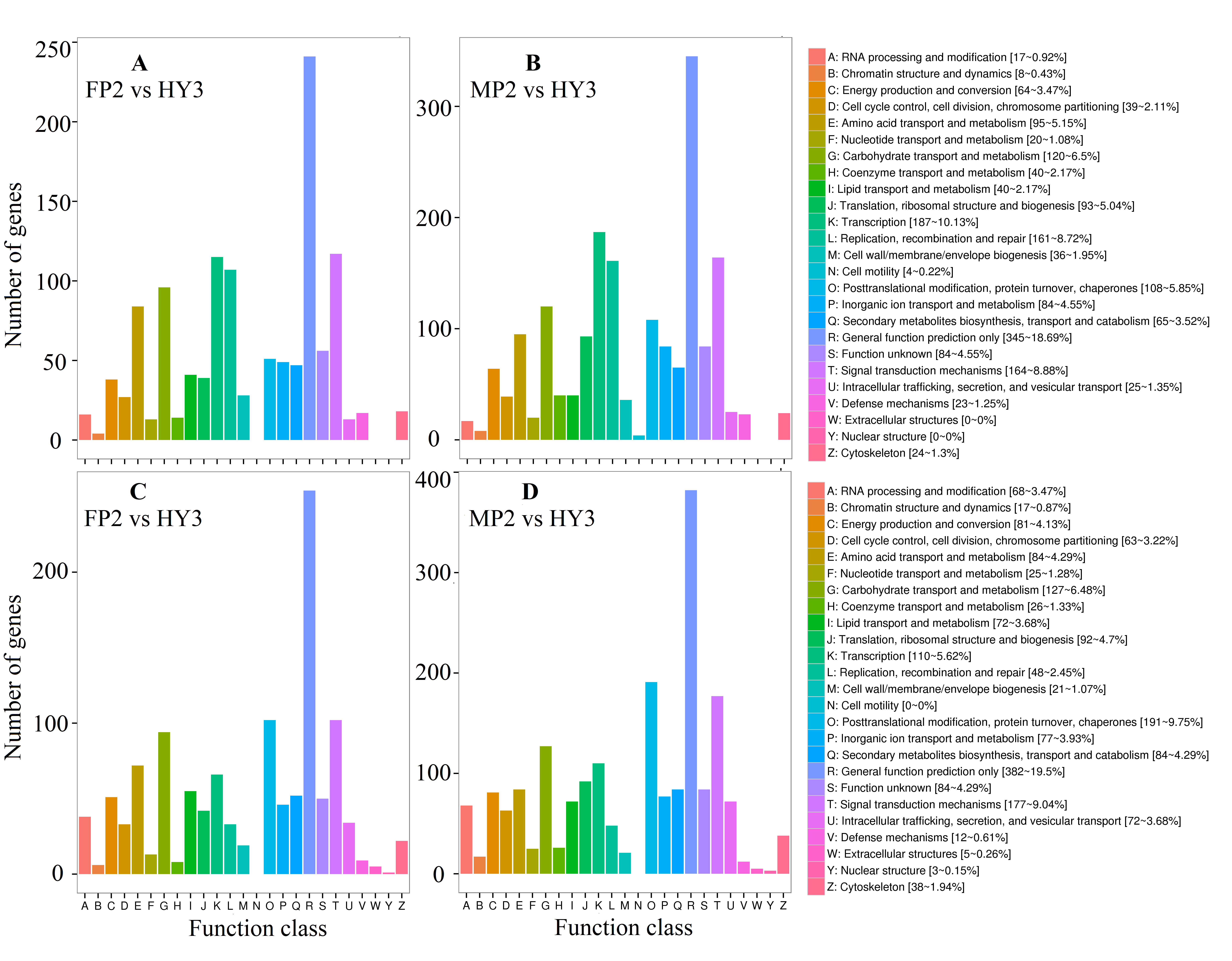

Supplement: Supplementary file 1 [file plants-10-00275-s001.zip › Supplementary materials/Figure S2.tif]

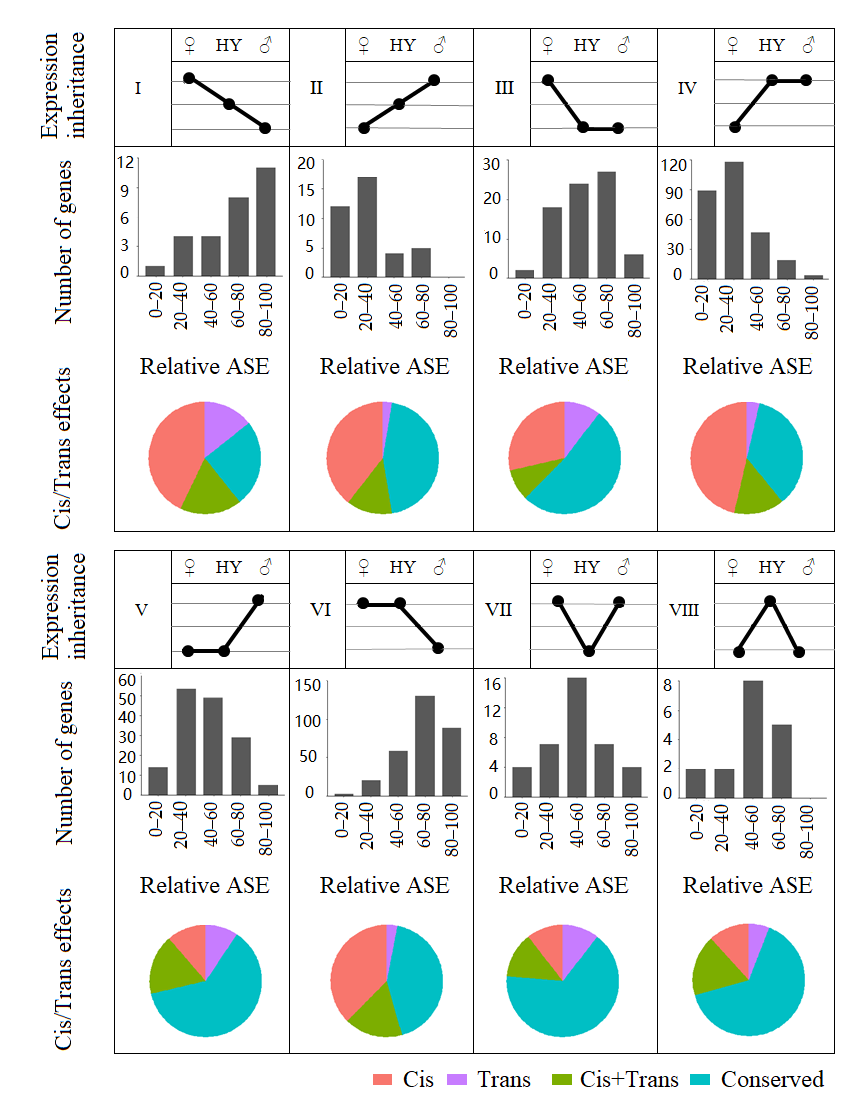

Supplement: Supplementary file 1 [file plants-10-00275-s001.zip › Supplementary materials/Figure S3.tif]

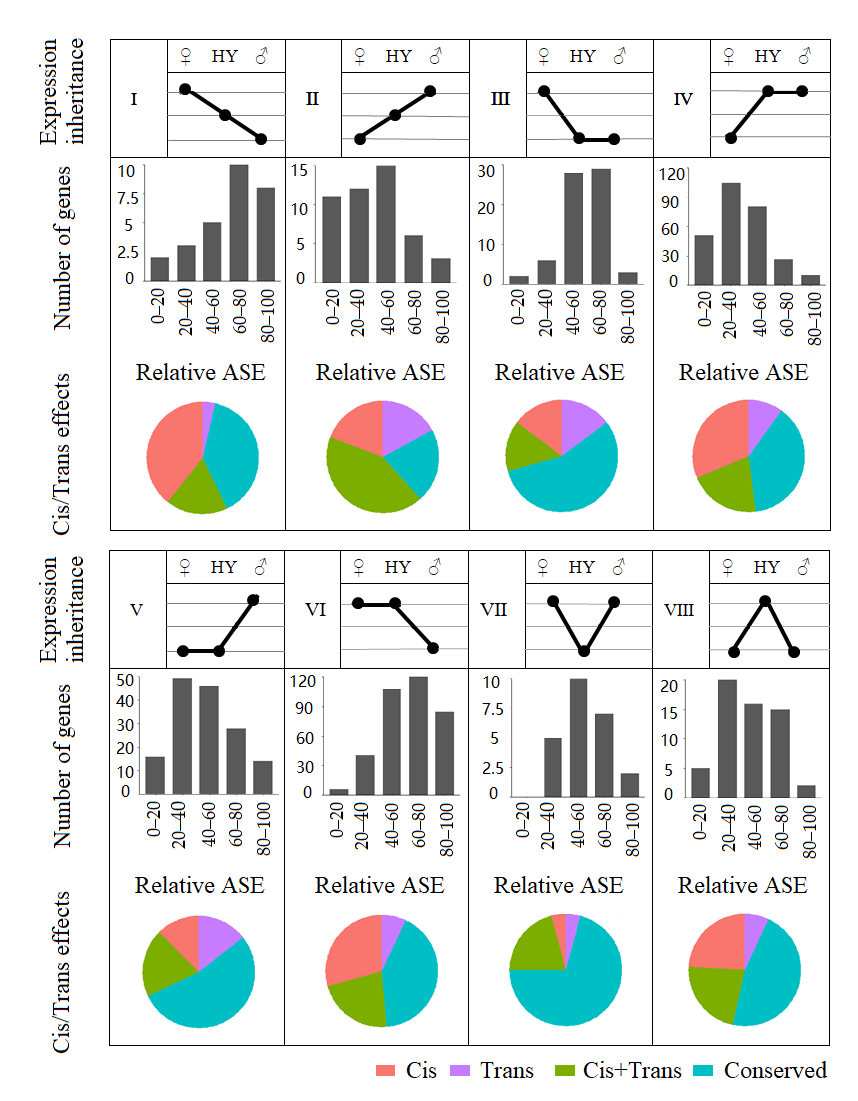

Supplement: Supplementary file 1 [file plants-10-00275-s001.zip › Supplementary materials/Figure S4.tif]

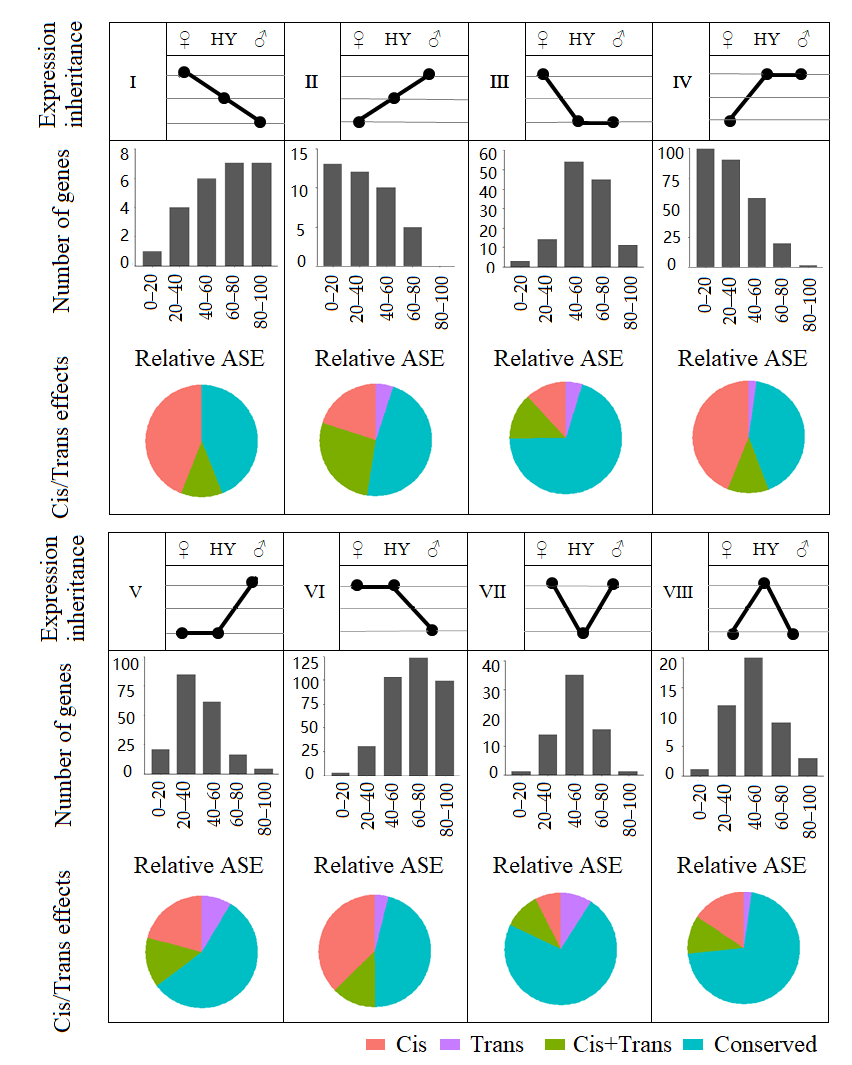

Supplement: Supplementary file 1 [file plants-10-00275-s001.zip › Supplementary materials/Figure S5.tif]

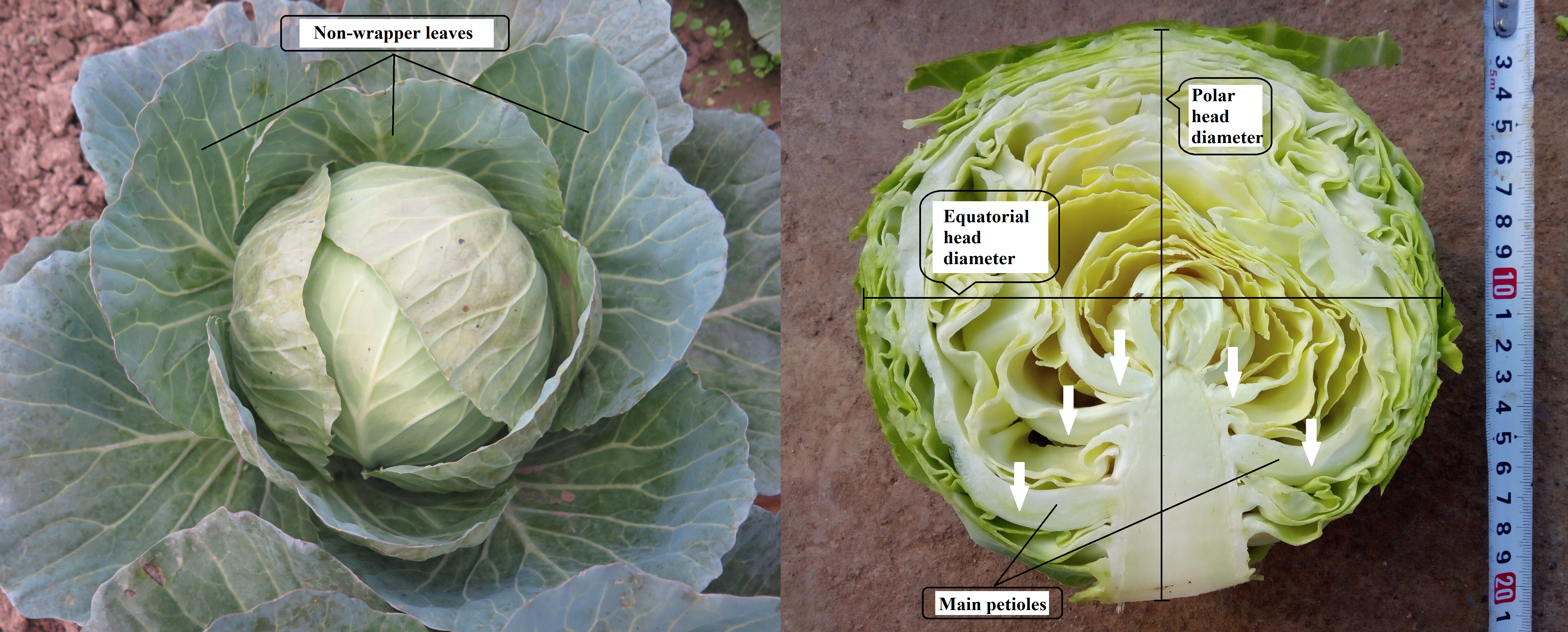

Supplement: Supplementary file 1 [file plants-10-00275-s001.zip › Supplementary materials/Figure S6.tif]
